# Current vehicle emission standards will not mitigate climate change or improve air quality

**DOI:** 10.1038/s41598-023-34150-7

**Published:** 2023-04-30

**Authors:** Andrew Jiaxuan Law, Ricardo Martinez-Botas, Phil Blythe

**Affiliations:** 1 Turbo Group, Department of Mechanical Engineering, Imperial College London, London, UK; 2 Future Mobility Group, School of Engineering, Newcastle University, Newcastle upon Tyne, UK

**Keywords:** Mechanical engineering, Climate-change mitigation

## Abstract

The vehicle emissions testing programme was conducted by the UK Department of Transport in 2016 in response to emissions tampering exposed in the Volkswagen (VW) emissions scandal. The programme identified large emissions discrepancies between real-world and in-lab testing across a range of Euro 5 and Euro 6 diesel passenger vehicles. The large vehicle test fleet reflects the current challenges faced in controlling vehicle emissions. This paper presents the following findings: NO_x_ emissions are altered due to exhaust gas recirculation mismanagement. A new Real-Life Emissions methodology is introduced to improve upon the current Real Driving Emissions standard. A large and concerning emissions divergence was discovered between the achieved NO_x_ improvement and deterioration of CO_2_. The findings act as catalysts to improve vehicle emissions testing beyond standards established since the VW scandal, aiding in the development of better climate change mitigation strategies and bring tangible air quality improvements to the environment.

## Introduction

The challenge of controlling global greenhouse gas (GHG) emissions is at a critical stage, where global warming has accelerated exponentially with record-high rates of CO_2_ production^1^. Control of CO_2_ levels is crucial in slowing down the depletion of the global carbon budget^2^ and preventing the irreversible global warming impact of 1.5 °C global temperature rise above pre-industrial levels^3,4^. As such, the need for large-scale global decarbonization has reached a level of high urgency^5^. In the UK, the transport sector stands as the largest contributor of domestic GHG emissions (CO_2_) at 27% in 2019, with cars and taxis occupying the largest proportion of 55% within transportation^6^. Furthermore, 50% of UK domestic NO_x_ emissions arises from road transport^7^. This is especially worrying for densely populated regions, where secondary gaseous emissions pose as direct harmful pollutants to those situated close to the road environment, and directly contributes to various respiratory and cardiovascular diseases^8,9^.

The Euro emission legislations stands as the current emissions standard for vehicle type approvals in the European Union (EU), with Euro 5 and 6 being the current limits in the UK^10^. Moreover, Euro 7 represents the final and most stringent emissions standard commencing from 2025^11,12^. Euro 7 will work alongside a combination of regional regulations, such as the Ultra-Low Emissions Zone (ULEZ) which only allows Euro 6 or better vehicles to operate within Central London^13,14^, and more widely the Clean Air Zones^15^ being introduced across UK cities. Future efforts towards a greener transport sector include ending fossil fuel vehicle purchases by 2030, and all new vehicles must operate with zero tailpipe emissions by 2035^16^. These strategies represent milestones towards large-scale road transport decarbonization in the UK^17^ which is encompassed in the Department for Transport (DfT) Transport Decarbonisation Plan^18^, and advancing towards net-zero carbon by 2050^19,20^.

However, pursuing carbon depletion through pure electrification is insufficient to meet the desired decarbonization targets in transportation^21^, as existing internal combustion engine vehicles (ICEVs) can still be utilized beyond the 2030 target until they are fully banned on UK roads, and can be utilized in third world countries for many years after that. As such, it is imperative to ensure that current emission testing protocols towards ICEVs are still being reassessed with stringency. Unfortunately, the recently phased out legislative New European Driving Cycle (NEDC) test was shown to be inadequate in reflecting the true real-world emission performances of ICEVs. This was specifically addressed under the Vehicle Emissions Testing Programme conducted by the DfT in 2016^22,23^ in response to the Volkswagen emissions scandal^24^. It demonstrated evidence of vehicle emissions tampering through software installation which detected the NEDC test cycle and ‘gamed’ it. Data of the testing programme is freely available under the gov.uk website^22,23^. This paper analyzes the results of 3 tests that were conducted within the programme: the in-lab NEDC^25^ (NEDC hot), NEDC on track conditions (NEDC track)^22,23^, and the Real Driving Emissions (RDE) test^26^, with the focus being on NO_x_ and CO_2_ emissions from the diesel passenger vehicles. Based on these results, this paper assesses Exhaust Gas Recirculation (EGR) mismanagement during emissions testing, proposing a stricter Real Life Emissions (RLE) assessment method, and analysing the divergence between NO_x_ and CO_2_ emissions.

## Exhaust gas recirculation mismanagement

**Figure 1:**
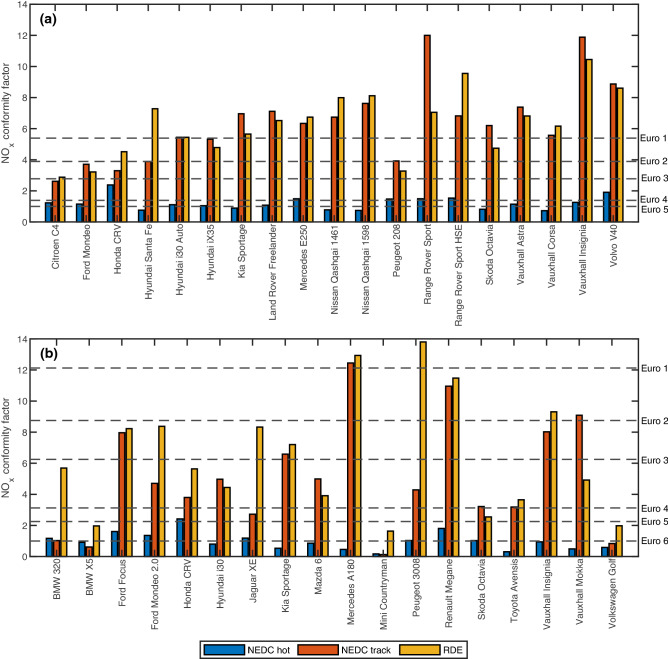
NO_x_ conformity factors of tested vehicles in NEDC hot, NEDC track and RDE tests. (**a**,**b**) Euro 5 vehicles (**a**), Euro 6 vehicles (**b**). The conformity factor is defined as the ratio of the recorded NO_x_ emission rate to the Euro limit of the vehicle. A factor value of 1 implies that the vehicle is abiding towards the emission limit. Bar heights represents the conformity factor values, and dashed lines represent the limits from Euro 1 to Euro 6 (note that the true Euro 1 and Euro 2 limits includes NO_x_ and unburnt hydrocarbons emissions). The number of vehicles that failed to meet the different Euro limits in RDE testing are as follow: For (**a**) —Euro 1: 13, Euro 2: 16, Euro 3 and above: 19. For (**b**) —Euro 1: 2, Euro 2: 4, Euro 3: 8, Euro 4: 14, Euro 5: 15, Euro 6: 18.

The Vehicle Emissions Testing Programme delivered an independent assessment to identify software tampering strategies in passenger vehicles. A range of Euro 5 and 6 vehicles were chosen to encapsulate 75% of sales of the top 70 vehicles in the UK. This selection represents more than 50% of all diesel passenger cars models licensed and in use on UK roads^22^. Although no evidence of software tampering was found (apart from the VW case), major differences between in-lab and real-world emissions performances were exhibited. Figure 1 displays the various NO_x_ emissions results of the Euro 5 (< 180 mg/km) and Euro 6 (< 80 mg/km) diesel vehicle fleets across the three tests: NEDC hot, NEDC track and RDE. Results are expressed using the conformity factor (CF), defined as the ratio of the recorded emission value to its Euro limit. Although the vehicles conformed to their legislative limits in laboratory testing, emission levels are significantly higher in real world testing conditions. A large majority produced a CF value greater than 2 in either NEDC track or RDE testing and substantially exceeded this value. More specifically, the RDE tests revealed that around half of the Euro 6 fleet were only Euro 3 compliant on the road (< 500 mg/km, imposed in 2000^27^), and most Euro 5 vehicles were not even Euro 1 compliant (< 970 mg/km, imposed in 1992^28^). This raises concerns on the true real-world vehicle emission performances, their present compliance towards the Euro regulations even as of today in 2022, and the impact this may have on emissions modelling.

Moreover, the NEDC track test follows the same NEDC speed-time profile but driven on a track to remove effects of in-lab conditions. One would expect a reasonably close agreement in emissions results between the two tests, but Fig. 1 displays major differences across the two tests. Several factors account for the emissions discrepancies. These include inaccurate in-lab aerodynamics replication (from the dynamometer coast down value setting), flexibility in vehicle testing configurations to obtain the best manufacturer emission results, presence of real-world cornering effects in track, and differences in ambient conditions (mainly temperature)^22^. However, even considering these factors, the differences in CF values are too large. More surprisingly, many vehicles performed similarly in both NEDC track (a 20 min drive cycle) and RDE (90 min of flexible on-road driving) tests. This led to the main hypothesis of analyzing the impact of ambient testing temperature in affecting emissions performances due to its effect on EGR activation within a vehicle.

**Figure 2:**
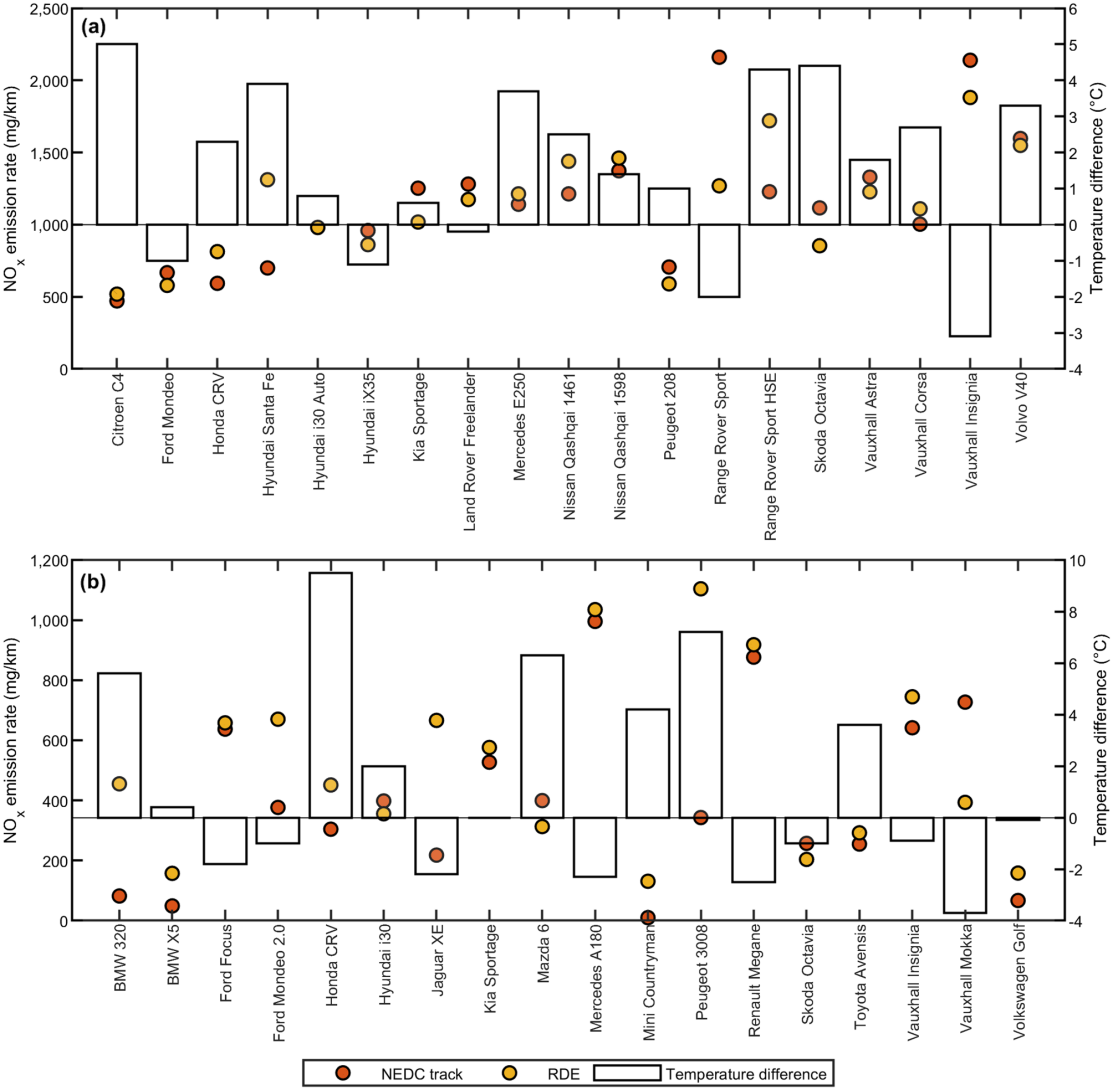
NO_x_ emission rates of tested vehicles in NEDC track and RDE tests, alongside the respective differences in ambient testing temperature between the two tests. (**a**,**b**) Euro 5 vehicles (**a**), Euro 6 vehicles (**b**). Circular points represent the NO_x_ emission rates. Bar heights represent the temperature difference, which is defined as the NEDC track test temperature minus the RDE test temperature.

Utilizing an EGR system reduces the production of NO_x_ emissions. However, limiting its activation under low ambient temperatures is a common practice amongst manufacturers to prevent moisture condensation and deposit build-up within the EGR system^22^. This temperature dependent strategy is implemented to protect against engine degradation and maximize vehicle operating lifetime. To assess the validity of the EGR temperature strategy, Fig. 2 compares the emission rates in both NEDC track and RDE tests based on the differences in ambient testing temperature. White bars indicate that the NEDC track test temperature was higher than the RDE test temperature, and grey bars indicate otherwise. Given that the EGR is expected to deactivate under colder testing temperatures, one would expect that NEDC track emission points (orange) should be below RDE points (yellow) upon having a higher NEDC track testing temperature, and the reverse should also hold true. However, many vehicles do not exhibit this trend.

To identify a considerable change in emissions performance and hence EGR manipulation, this work has set an emission difference (${\Delta }NO_{x}$) of 100 mg/km and above to be of significance, as changes larger than this implies that the vehicle’s emissions is operating under a different vehicle class (difference between Euro 5 and Euro 6), which is not acceptable. Defining temperature difference as ${\Delta }T$, vehicles that did not abide by the expected EGR-temperature strategy were categorized under 3 anomalies. The first anomaly displays the wrong temperature-emission correlation, whereby the NO_x_ emission rate is lower in the colder test. The second anomaly represents a large change in ambient testing temperature (${\Delta }T \ge 1\;^\circ {\text{C}}$) yet changes in NO_x_ emissions are small (${\Delta }NO_{x} \le 100$ mg/km). Finally, the last anomaly is opposite to the second, whereby ${\Delta }T \le 1\;^\circ {\text{C}}$ but ${\Delta }NO_{x} \ge 100$ mg/km. Table 1 lists the vehicles which demonstrates the respective anomalies.

**Table 1:** Identification of vehicles exhibiting anomalies in the EGR temperature strategy.

	Description of anomalies		
	Wrong temperature-emission correlation	$\Delta T\ge 1^\circ \mathrm{C}$ but $\left|\Delta N{O}_{x}\right|\le 100 \mathrm{mg}/\mathrm{km}$ EGR deactivation not located in $\Delta T$ band	$\Delta T\le 1^\circ \mathrm{C}$ but $\left|\Delta N{O}_{x}\right|\ge 100 \mathrm{mg}/\mathrm{km}$ EGR deactivation located in $\Delta T$ band
***Euro 5 vehicles***			
Citroen C4		+	
Ford Mondeo		+	
Hyundai iX35		+	
Kia Sportage	+		+
Land Rover Freelander			+
Mercedes E250		+	
Nissan Qashqai 1598		+	
Peugeot 208	+		+
Skoda Octavia	+		
Vauxhall Astra	+		
Volvo V40	+	+	
***Euro 6 vehicles***			
BMW X5			+
Ford Focus	+	+	
Ford Mondeo 2.0	+		+
Hyundai i30	+	+	
Jaguar XE	+		
Mazda 6	+	+	
Mercedes A180	+	+	
Renault Megane	+	+	
Skoda Octavia		+	
Toyota Avensis		+	
Vauxhall Insignia	+		+
Volkswagen Golf	+		

Cells with + represent positive display of the associated anomaly.

Vehicles under the first anomaly represent the most concerning group as the temperature variation does not produce the expected change in NO_x_ emissions. This requires further investigation on a per vehicle basis. The second anomaly shows that the EGR deactivation temperature is not located within the $\Delta T$ band despite undergoing large temperature changes. This indicates that the EGR deactivation point is prescribed at either a colder temperature, or the EGR system has already been activated at a hotter temperature and NO_x_ emissions cannot be further decreased. Finally, the third anomaly shows that the EGR deactivation point is ideally located within the small $\Delta T$ band, which explains the large change in NO_x_ emissions.

The presence of these three anomalies further reinforces the wide variation in EGR strategies adopted across manufacturers and its impact in altering on-road NO_x_ emissions. Moreover, a recent ruling by the EU Court of Justice has prohibited the disabling of such defeat devices for the purpose of minimizing engine ageing and degradation^29^. This represents a momentous first step towards including the consideration for EGR mismanagement in future emissions testing to enforce greater responsibility and liability from vehicle manufacturers. However, note that vehicles listed in Table 1 are not solely at fault, as Fig. 1 still shows that all vehicles were not compliant to the Euro limits under real world testing, regardless of considering EGR mismanagement. Other factors are also in play, and this requires further examination on a vehicle manufacturer case-by-case basis.

## Real Life Emissions (RLE)

The RDE test successfully highlighted differences between in-lab and real-world emission values. However, the RDE drive cycle profile is legislated to contain approximately 60% of the time spent in urban driving and 40% in motorway driving^23^. This proportion split does not reflect the driving pattern of every passenger, given that there are numerous factors that influence daily travel patterns (such as geography, gender and age, trip purpose etc^30^). While the RDE test has brought forward a much-needed level of transparency in emissions testing compared to in-lab methods, further improvements can be made. More specifically, there is a need to scale real-world emissions results from the fixed RDE drive cycle and apply it to different urban-motorway time proportions. Therefore, the Real Life Emissions (RLE) method was conceived in this paper to evaluate changes in emission levels with varying cycle compositions, and the RLE evaluates a new combined emission rate based on the selected time proportions. Derivation of the RLE is described in the Methods section.

**Figure 3:**
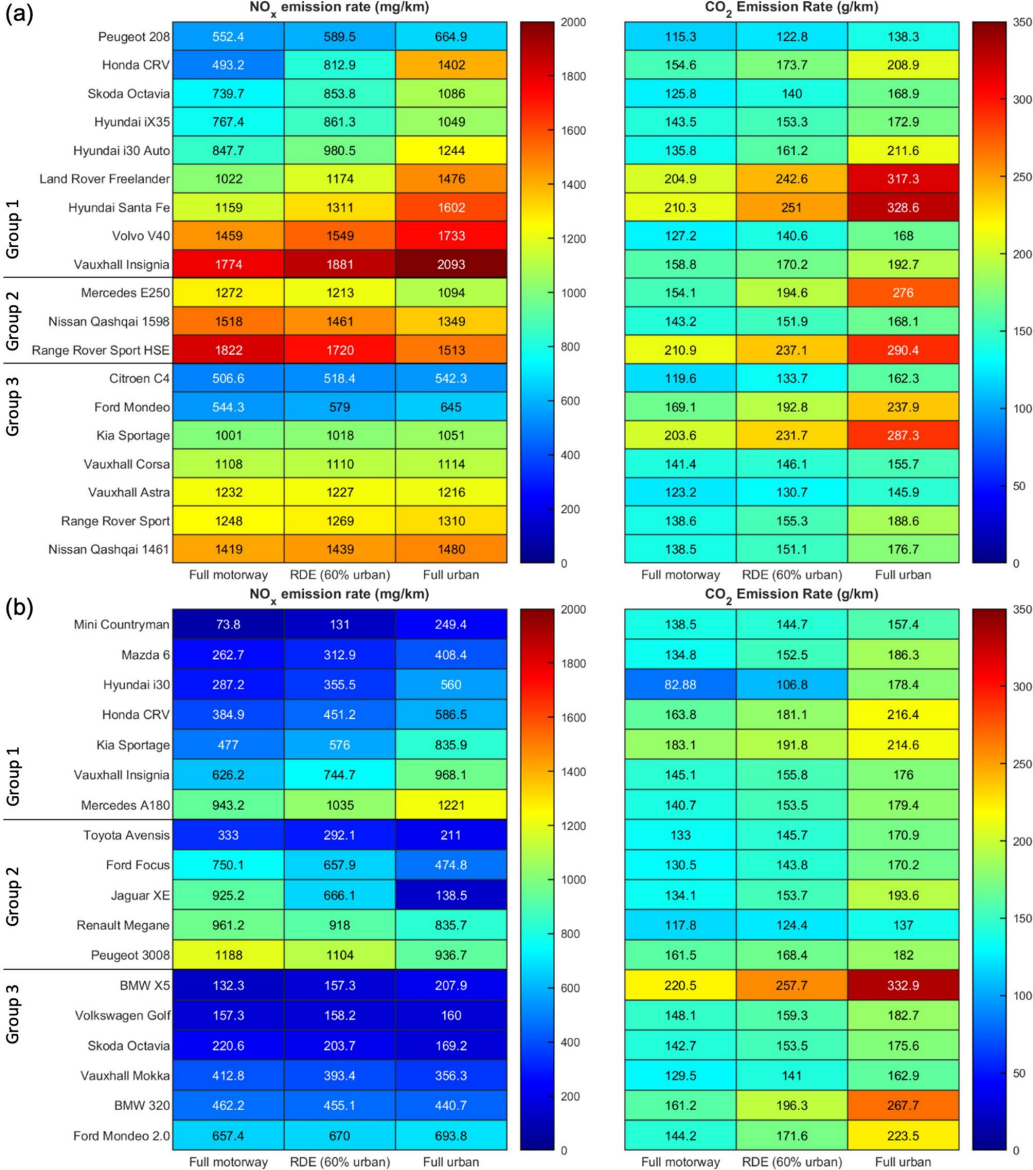
NO_x_ and CO_2_ emission rates of the tested vehicles based on the RLE methodology, with emission values presented in full urban, RDE (60% urban) and full motorway driving compositions. (**a**,**b**) Euro 5 vehicles (**a**), Euro 6 vehicles (**b**). Emission rates are represented by a heat map, with dark blue representing zero emissions and dark red representing the highest emission rate. For NO_x_, the vehicles are further arranged into 3 groups: Group 1—Increasing NO_x_ emissions with greater urban driving, Group 2—Decreasing NO_x_ emissions with greater urban driving, Group 3—NO_x_ emissions being independent of drive cycle composition. Plots for the NO_x_ and CO_2_ full emissions spectrums across varying time proportions are available in Supplementary Information.

Figure 3 displays the RLE results of NO_x_ and CO_2_ emission rates for each tested vehicle, with values presented at full motorway, RDE (60% urban), and full urban driving compositions. For NO_x_ emissions, it is evident that every vehicle contains a different emissions response with progression from full motorway to full urban driving. Three main groups can be classified based on their respective NO_x_ emissions trends: Group 1—Increasing NO_x_ emissions with greater urban driving, Group 2—Decreasing NO_x_ emissions with greater urban driving, and Group 3—NO_x_ emissions being relatively independent of drive cycle composition. Again, the same difference of ${\Delta }NO_{x} \ge 100$ mg/km was chosen to reflect significant change in emissions. Group 1 vehicles pose an immediate health hazard towards citizens close to the roadside due to higher NO_x_ emissions in urban conditions where direct NO_x_ exposure is of concern. In contrast, Group 2 vehicles contain higher motorway emission rates. Combined with longer travelling distances in motorway driving, such vehicles will produce greater cumulative NO_x_ amounts. Finally, emission performances from Group 3 vehicles are representative of any driving scenario, as the NO_x_ emission rates are approximately constant regardless of cycle composition.

For CO_2_ emissions, Fig. 3 demonstrates a consistent increase in emission rate with greater urban driving across all vehicles. This is associated to the sustained power draw during start-stop urban driving alongside the shorter distance coverages, hence inflating CO_2_ production per km. Although this negatively portrays vehicle driving under pure urban conditions, the lower motorway emission rates will be countered by the significantly longer travelling distances. Driving mileage still stands as the most important factor in determining the final cumulative CO_2_ production from passenger vehicles.

Variances in NO_x_ and CO_2_ emission spectrums across different urban-motorway driving proportions accentuates the importance of integrating the RLE method, instead of solely relying on a single drive cycle composition result. Evaluation of air quality depends on accurate emissions modelling known as vehicle emission factors^31^. The RLE method helps to categorize vehicles based on their respective NO_x_ emissions trends. These can then be applied on a local area basis to improve emissions factor modelling and bring better air quality policing. This method can also be similarly applied to improve predictions on cumulative emissions modelling across a nationwide scale^32,33^, which helps to enforce more impactful legislations targeted towards combating the finite global carbon budget.

## Divergence in vehicle emissions

**Figure 4:**
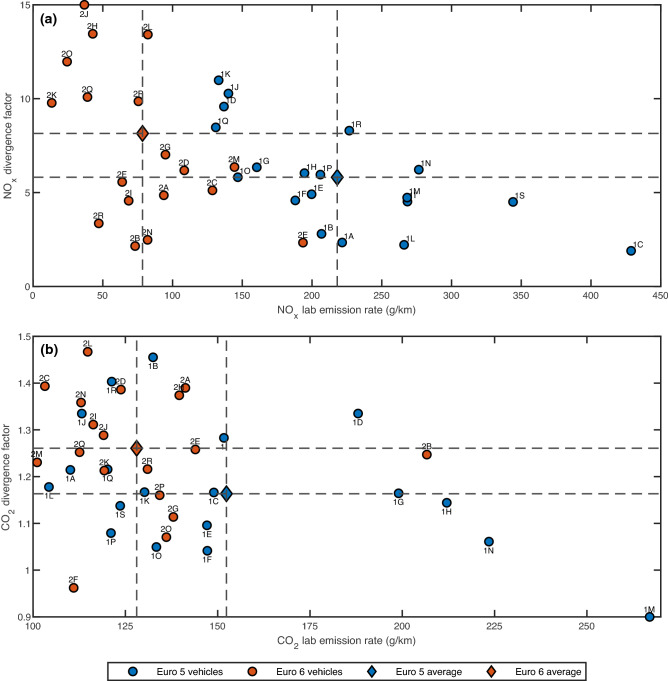
Development of NO_x_ and CO_2_ Lab-to-RDE divergence ratio from the Euro 5 to Euro 6 vehicle fleet. (**a**,**b**) NO_x_ divergence factor (**a**), CO_2_ divergence factor (**b**).The divergence factor is defined as the ratio of the RDE emission rate to the in-lab NEDC emission rate. Circular scatter points represent the divergence factor corresponding to the in-lab emission rate, and each point is denoted by the label of each tested vehicle as listed in Table 2. Advancing from the blue to orange diamond scatter point represents the development of the average divergence ratio moving from the Euro 5 to Euro 6 fleet. Note that the true points for 2 J in (**a**) and 1 M in (**b**) are located far outside of the axis limits. They are located at (36.8 mg/km, 28.1) and (267.7 g/km, 0.580) respectively.

The current CO_2_ target in the EU involves limiting vehicle emissions of new passenger cars to a fleet-wide average of 95 gCO_2_/km from 2020^34^. However, unlike the Euro standards, there is no quantitative limit on tailpipe CO_2_ emissions for each individual vehicle. As such, the divergence factor was proposed to assess the increase in CO_2_ emission rate from the in-lab NEDC hot test to the real-world RDE test. NO_x_ divergence factors (ratio of RDE NO_x_ to in-lab NEDC NO_x_ emission rate) were also calculated to assess the NO_x_ variance within each Euro fleet. Figure 4 shows the development of both CO_2_ and NO_x_ divergence factors from Euro 5 to Euro 6, reflecting the progression towards stricter emission limits from 2011 to 2016^10^ and beyond, and Table 2 contains the list of labels for the Euro 5 and 6 vehicles in Fig. 4.

**Table 2:** Labelling of Euro 5 and Euro 6 vehicles for Fig. 4.

Euro 5 fleet		Euro 6 fleet	
Label	Vehicle	Label	Vehicle
1A	Citroen C4	2A	BMW 320
1B	Ford Mondeo	2B	BMW X5
1C	Honda CRV	2C	Ford Focus
1D	Hyundai Santa Fe	2D	Ford Mondeo 2.0
1E	Hyundai i30 Auto	2E	Honda CRV
1F	Hyundai iX35	2F	Hyundai i30
1G	Kia Sportage	2G	Jaguar XE
1H	Land Rover Freelander	2H	Kia Sportage
1I	Mercedes E250	2I	Mazda 6
1J	Nissan Qashqai 1461	2J	Mercedes A180
1K	Nissan Qashqai 1598	2K	Mini Countryman
1L	Peugeot 208	2L	Peugeot 3008
1M	Range Rover Sport	2M	Renault Megane
1N	Range Rover Sport HSE	2N	Skoda Octavia
1O	Skoda Octavia	2O	Toyota Avensis
1P	Vauxhall Astra	2P	Vauxhall Insignia
1Q	Vauxhall Corsa	2Q	Vauxhall Mokka
1R	Vauxhall Insignia	2R	Volkswagen Golf
1S	Volvo V40		

Figure 1 shows that the average NO_x_ conformity factor for the RDE test maintained at approximately 6.4 while progressing from Euro 5 to Euro 6, an improved (but still insufficient) reduction in real-world NO_x_ emissions. However, Fig. 4 shows that the average NO_x_ divergence has increased from Euro 5 at 5.81 to Euro 6 at 8.15, demonstrated by the wider vertical spread within the Euro 6 fleet. This is an undesirable outcome as the goal towards lowering NO_x_ emissions hinders the ability to enforce emissions control uniformity across a fleetwide basis. On the CO_2_ side, real world CO_2_ emission rates from both vehicles fleets are similar (as observed in Fig. 3), but the progression from Euro 5 to Euro 6 also showed an increase in average divergence from 1.16 to 1.26, again indicating the larger unwanted CO_2_ emissions variance.

A study conducted by the International Council on Clean Transportation (ICCT) covering an enormous dataset of 1.3 million vehicles across eight European countries, demonstrated an average increase in CO_2_ divergence from 8% in 2001 to 39% in 2017^35^. These results coincide with the results above, with the testing programme focusing just on UK passenger vehicles^22^, but the ICCT study did not investigate divergence in NO_x_ emissions. On the other hand, various research has estimated NO_x_ divergence values of 6–7 compared to in-lab results ^36–38^ which concur with the findings in Figs. 1 and 4, but again lacking concurrent analysis of both CO_2_ and NO_x_ emissions. This paper presents an assessment of the increase in both NO_x_ and CO_2_ divergence from Euro 5 to Euro 6 vehicle fleet, showing the ineffectiveness of reducing greenhouse CO_2_ emission levels while placing an overemphasis on containing Euro emissions which are geared towards air quality. Despite advancing towards stricter Euro regulations, CO_2_ emission rates in both Euro 5 and 6 vehicles are similar, while both CO_2_ and NO_x_ variances have worsened. This calls for the need to emphasize the importance of future simultaneous control of both emission types.

## Discussion

The Vehicle Emissions Testing Programme demonstrated the inadequacy of the laboratory NEDC test in reflecting real-world emissions. Results from the NEDC track and RDE tests reveal real-world NO_x_ production from both Euro 5 and 6 vehicles are only Euro 3 compliant at best. The latest real-world regulatory standard RDE4 requires all new vehicles (1st January 2021 onwards) to abide towards a conformity factor of 1.43 or lower, with the 0.43 margin accounting for uncertainties in emissions measurements^26,39^. However, the tested vehicles clearly exceed this threshold, and future testing policies need to be sufficiently demanding to ensure real-world emissions are drastically improved upon moving towards Euro 7. Furthermore, certain vehicles did not exhibit the expected changes in NO_x_ emissions despite differences in ambient testing temperatures, and 3 main anomalies related towards the EGR-temperature activation strategy were highlighted to account for the observation. It was determined that EGR activation mismanagement stands as a prime driver in producing such varied NO_x_ emissions results, and EGR manipulation necessitates greater attention and consideration in future emissions testing.

The RLE method strengthens the effectiveness of the RDE test through the creation of emission spectrums with varying urban-motorway driving proportions. Its application further reveals differences in NO_x_ emissions performances, with vehicles performing better or worse in full urban conditions, or having stable emission rates that are independent of drive cycle composition. Moreover, increases in CO_2_ emission rates with greater urban driving was observed across all vehicles. The proposed RLE method could stand as an integral component for emissions modelling given its improved testing transparency and strong applicability towards any emissions standard. As implementation of emissions standards (such as the Clean Air Zones) relies on accurate modelling of the vehicle emission factors^31^, the RLE method could greatly reduce the modelling uncertainties. By accounting for variations in individual driving patterns and habits^30^ across regions, the RLE method can be integrated to implement effective emissions policing, bringing in tangible air quality improvements across environments scaling locally to nationwide.

Lastly, although the progression from Euro 5 to Euro 6 successfully reduced the average NO_x_ emission production, the divergence of both NO_x_ and CO_2_ emission rates has worsened. More importantly, the control of CO_2_ emissions has been neglected as real-world emission rates from both vehicle fleets are similar. As such, elevating awareness for the simultaneous improvement in both CO_2_ and NO_x_ emissions is imperative in hopes of prohibiting the same trend from occurring while advancing to the final Euro 7 standard in 2025. Additionally, this is pertinent towards low- or middle-income nations outside of the EU which are either on or advancing towards the Euro 5 or 6 emissions standards, and countries who utilizes second-hand vehicles from the UK. Prevention of this divergence must be undertaken.

In conclusion, the Vehicle Emissions Testing Programme has served as a foundation in displaying the inadequacy of the laboratory testing in portraying real-world emission levels. While the in-lab NEDC standard has been replaced by the Worldwide Harmonised Light Vehicles Test Procedure^25^ alongside RDE testing, further improvements in emissions testing need to be delivered in the form of EGR activation control, the RLE method, and emissions divergence assessment. Euro 5 and 6 vehicles are still prevalent in the ongoing transition towards transport decarbonization. As such, these methods stand as powerful, next-generation metrics to further improve vehicle emissions testing and reduce uncertainties in future emissions modelling for better climate mitigation policies. Integration of these in-depth emissions analysis will aid in the proposal of more impactful on-road emissions legislations and smarter traffic management, thus helping to avoid additional emissions-related premature deaths and push towards achieving the long-term goal of net-zero carbon by 2050.

## Methods

Data for the Vehicle Emissions Testing Programme is freely available from the gov.uk website^22,23^. Testing data are provided for the three tests discussed in this paper: NEDC hot, NEDC track and RDE, specifically dealing with data for CO_2_ emissions, NO_x_ emissions and ambient testing temperature.

The RLE methodology assess variations in the combined emission rate value upon changing the composition of the RDE drive cycle. Decomposition of the urban and motorway driving data was dependent on the respective drive cycle profiles. Urban driving consists of low driving speeds mainly under 48 km/h with multiple start-stop occurrences, whilst motorway driving consists of sustained periods of high-speed driving above 48 km/h. Upon separation of the urban and motorway portions, the average urban emission $k_{u}^{d}$ and average motorway NO_x_ emission $k_{m}^{d}$ per unit distance (g/km for CO_2_ or mg/km for NO_x_) can be determined by:

1 $$k_{u}^{d} = \frac{1}{{d_{u} }}\mathop \smallint \nolimits_{0}^{{t_{u} }} k_{u}^{t} dt$$

2 $$k_{m}^{d} = \frac{1}{{d_{m} }}\mathop \smallint \nolimits_{{t_{u} }}^{{t_{RDE} }} k_{m}^{t} dt$$

where $k$ represents either NO_x_ or CO_2_, $k_{u}^{t}$ and $k_{m}^{t}$ represents the urban and motorway emission per unit time respectively (g/s for CO_2_ and mg/s for NO_x_), $d_{u}$ and $d_{m}$ represents the urban and motorway distances respectively, and $t_{u}$ and $t_{RDE}$ represents the urban and the final RDE cycle time duration respectively. The motorway time duration $t_{m}$ is equal to $t_{RDE} - t_{u}$.

In an arbitrary driving cycle composition, the combined emission rate per unit distance $k_{c}^{d}$ can be expressed by the following equation:

3 $$k_{c}^{d} = k_{u}^{d} \times \frac{{d_{u} }}{{d_{RDE} }} + k_{m}^{d} \times \frac{{d_{m} }}{{d_{RDE} }}$$

where $d_{RDE}$ is the total RDE driving distance. Defining the urban and motorway distance proportions as $D_{u}$ and $D_{m}$:

4 $$D_{u} \equiv \frac{{d_{u} }}{{d_{RDE} }},D_{m} \equiv \frac{{d_{m} }}{{d_{RDE} }}$$

5 $$D_{u} + D_{m} = 1$$

6 $$\therefore k_{c}^{d} = k_{u}^{d} \times D_{u} + k_{m}^{d} \times D_{m}$$

However, using time proportions spent in urban and motorway driving ($T_{u}$ and $T_{m}$) serves as a better representation towards characterizing the driving habits of individual passengers:

7 $$T_{u} \equiv \frac{{t_{u} }}{{t_{RDE} }}, T_{m} \equiv \frac{{t_{m} }}{{t_{RDE} }},$$

8 $${\text{and }}T_{u} + T_{m} = 1$$

Therefore, it is more appropriate to express the two distance proportions as functions of their individual time proportions, where $D_{u/m} = f\left( {t_{u/m} } \right)$. Derivation of the respective functions are as follow:

9 $$D_{u} \equiv \frac{{d_{u} }}{{d_{RDE} }} = \frac{{d_{u} }}{{d_{u} + d_{m} }} = \frac{{t_{u} \times V_{u} }}{{t_{u} \times V_{u} + t_{m} \times V_{m} }} = \frac{{t_{u} \times V_{u} }}{{t_{u} \times V_{u} + \left( {t_{RDE} - t_{u} } \right) \times V_{m} }}$$

where $V_{u}$ and $V_{m}$ represents the average urban and motorway speed of the drive cycle respectively. Dividing Eq. (9) by $t_{RDE}$:

10 $$\begin{aligned} D_{u} & = \frac{{t_{u} /t_{RDE} \times V_{u} }}{{t_{u} /t_{RDE} \times V_{u} + \left( {1 - t_{u} /t_{RDE} } \right) \times V_{m} }} \\ \therefore D_{u} \left( {T_{u} } \right) & = \frac{{T_{u} }}{{T_{u} + (1 - T_{u} ) \times V_{m} /V_{u} }} \\ \end{aligned}$$

Finally, using Eq. (5) to calculate $D_{m}$:

11 $$\therefore D_{m} \left( {T_{u} } \right) = 1 - D_{u} \left( {T_{u} } \right) = \frac{{1 - T_{u} }}{{T_{u} \times V_{u} /V_{m} + (1 - T_{u} )}}$$

and $k_{c}^{d}$ can be expressed purely as a function of $T_{u}$ (or $T_{m}$):

12 $$\therefore k_{c}^{d} \left( {T_{u} } \right) = k_{u}^{d} \left[ {\frac{{T_{u} }}{{T_{u} + (1 - T_{u} ) \times V_{m} /V_{u} }}} \right] + k_{m}^{d} \left[ {\frac{{1 - T_{u} }}{{T_{u} \times V_{u} /V_{m} + (1 - T_{u} )}}} \right]$$

Therefore, the combined emission rate per unit distance is also dependent on the average urban-to-motorway speed ratio $V_{u} /V_{m}$, which is a characteristic specific to the selected drive cycle. This methodology can then be applied similarly to other legislative drive cycles with knowledge of the average speeds of the two driving modes. Plots for the CO_2_ and NO_x_ combined emission rate of each vehicle in the range $T_{u} \in \left[ {0,1} \right]$ are available in the Supplementary Information (Supplementary Figs. 1–11).

## Supplementary Information

Supplementary Information.

## Data Availability

The datasets generated and/or analysed during the current study are available in the Vehicle Emissions Testing Programme: Data and Conclusions website that is provided by the UK Department of Transport, https://www.gov.uk/government/publications/vehicle-emissions-testing-programme-conclusions.
