# Supplementary material for: Current vehicle emission standards will not mitigate climate change or improve air quality
Source: Sci Rep. 2023 Apr 30;13:7060. doi: 10.1038/s41598-023-34150-7 (PMC10149503; doi:10.1038/s41598-023-34150-7)
Supplement: Supplementary file 1 — Supplementary Information. [file 41598_2023_34150_MOESM1_ESM.pdf]

Supplementary Information for Current vehicle emission standards will not mitigate climate change or improve air quality.

The following Supplementary Figures displays the Real Life Emissions (RLE) graphs of NO_x and CO_2 emission rates for the tested Euro 5 and 6 vehicles.

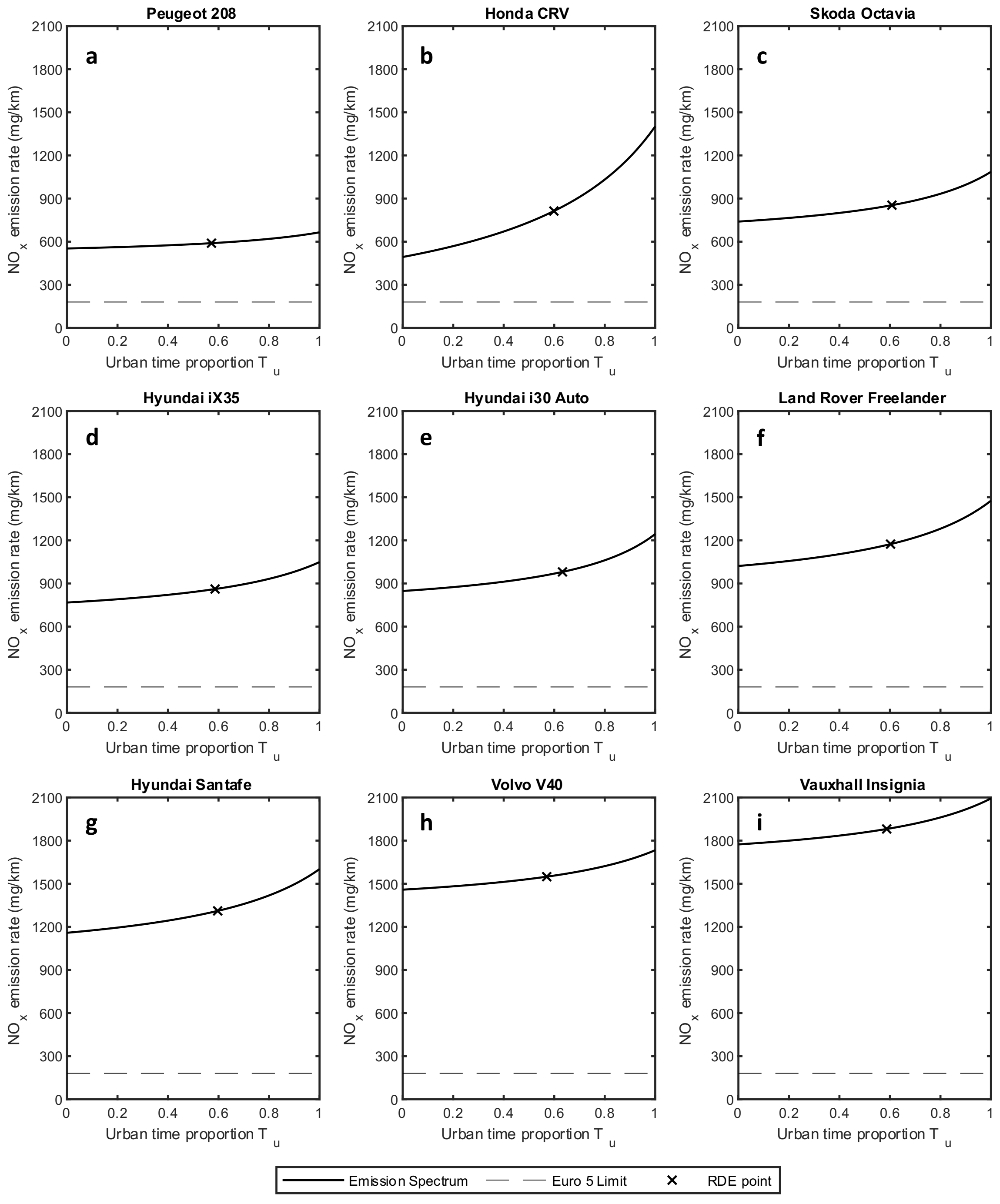

Supplementary Fig. 1 – RLE NO_x emissions spectrum for Euro 5 Vehicles under Group 1: Increasing NO_x emission rate with greater urban time proportion. a-i, Peugeot 208 (**a**), Honda CRV (**b**), Skoda Octavia (**c**), Hyundai iX35 (**d**), Hyundai i30 Auto (**e**), Land Rover Freelander (**f**), Hyundai Santafe (**g**), Volvo V40 (**h**), Vauxhall Insignia (**i**). The solid lines represent variation of the NO_x emission rate with increasing urban time proportion (for $T_u \in [0,1]$), the dashed lines represent the Euro 5 NO_x limit at 180 mg/km, and the cross-scatter points represent the recorded value at the RDE time proportion, where $T_u \approx 0.6$.

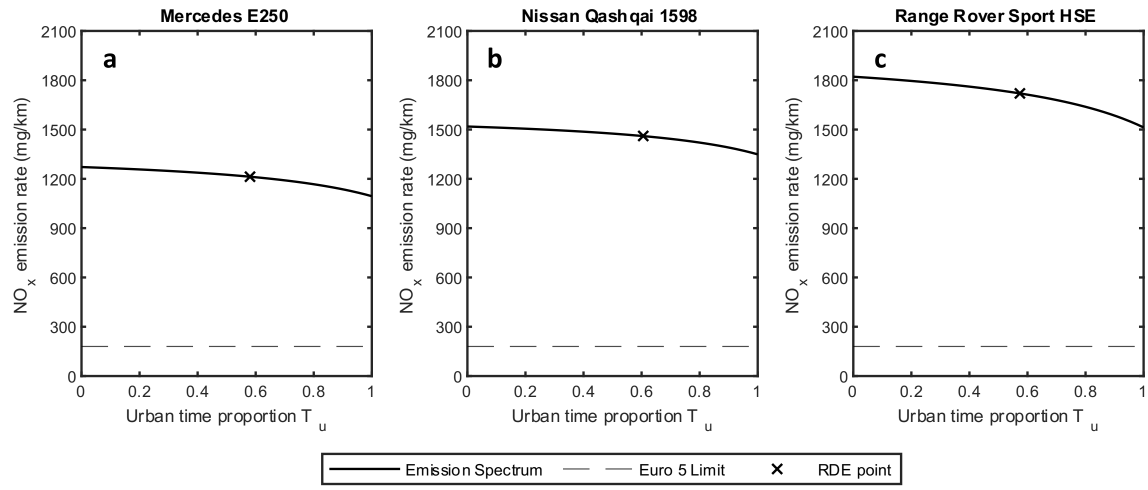

Supplementary Fig. 2 – RLE NO_x emissions spectrum for Euro 5 Vehicles under Group 2: Decreasing NO_x emission rate with greater urban time proportion. a-c, Mercedes E250 (**a**), Nissan Qashqai 1598 (**b**), Range Rover Sport HSE (**c**). The solid lines represent variation of the NO_x emission rate with increasing urban time proportion (for $T_u \in [0,1]$), the dashed lines represent the Euro 5 NO_x limit at 180 mg/km, and the cross-scatter points represent the recorded value at the RDE time proportion, where $T_u \approx 0.6$.

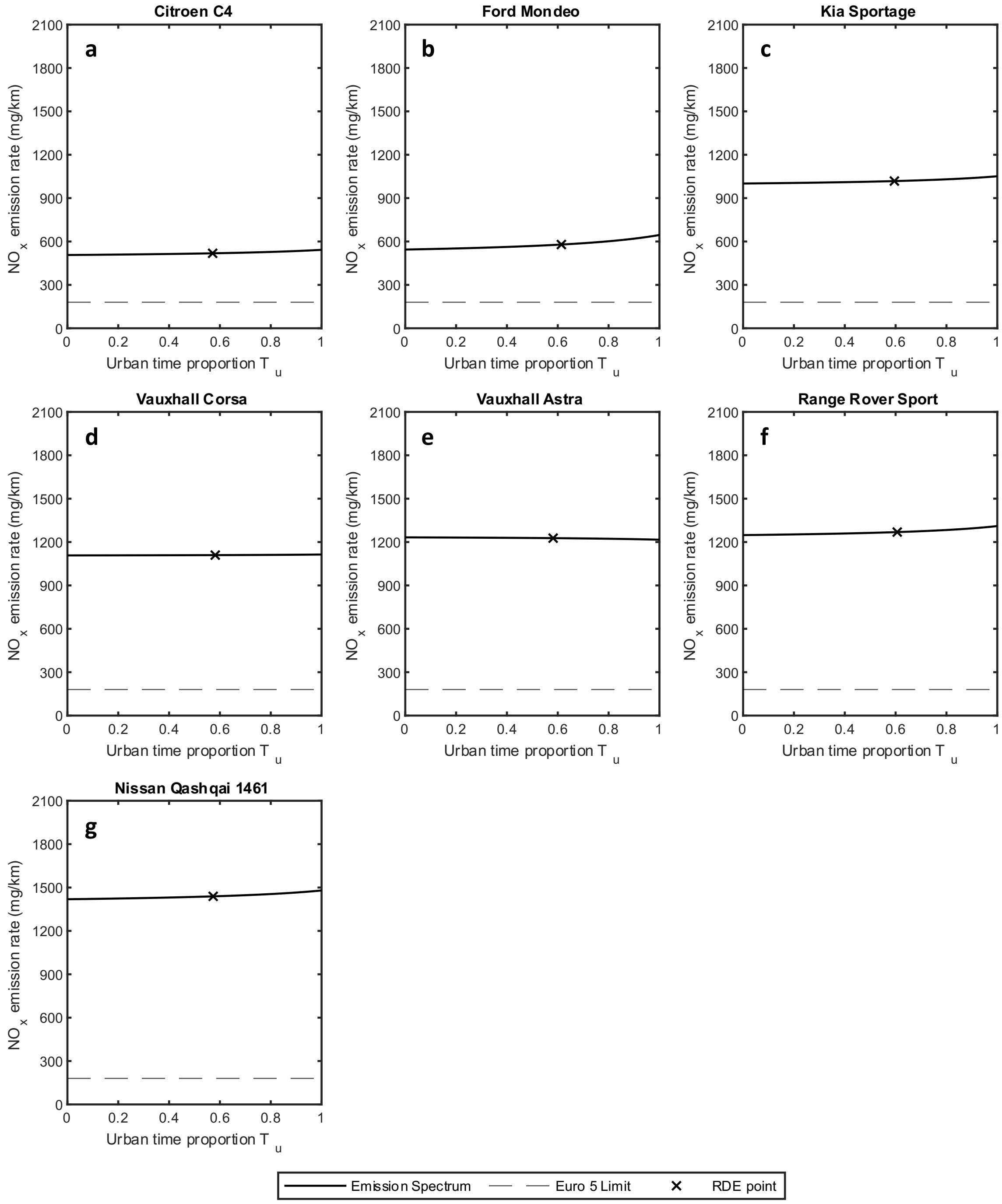

Supplementary Fig. 3 – RLE NO_x emissions spectrum for Euro 5 Vehicles under Group 3: NO_x emission rate independent of urban time proportion (with less than 100 mg/km difference). a-g, Citroen C4 (**a**), Ford Mondeo (**b**), Kia Sportage (**c**), Vauxhall Corsa (**d**), Vauxhall Astra (**e**), Range Rover Sport (**f**), Nissan Qashqai 1461 (**g**). The solid lines represent variation of the NO_x emission rate with increasing urban time proportion (for $T_u \in [0,1]$), the dashed lines represent the Euro 5 NO_x limit at 180 mg/km, and the cross-scatter points represent the recorded value at the RDE time proportion, where $T_u \approx 0.6$.

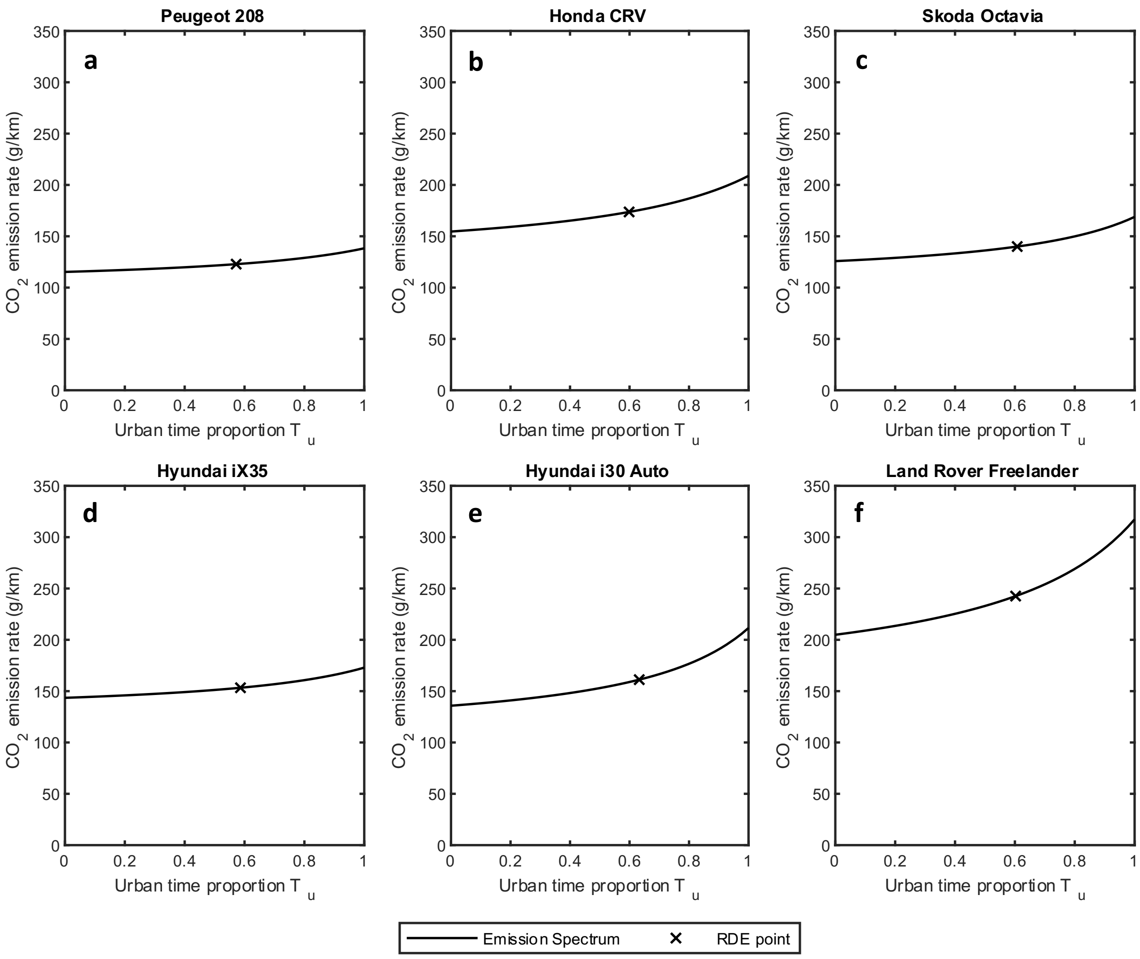

Supplementary Fig. 4 – RLE CO_2 emissions spectrum for Euro 5 Vehicles (Part 1). a-f, Peugeot 208 (**a**), Honda CRV (**b**), Skoda Octavia (**c**), Hyundai iX35 (**d**), Hyundai i30 Auto (**e**), Land Rover Freelander (**f**). The lines represent variation of the CO_2 emission rate with increasing urban time proportion (for $T_u \in [0,1]$), and the cross-scatter points represent the recorded value at the RDE time proportion, where $T_u \approx 0.6$.

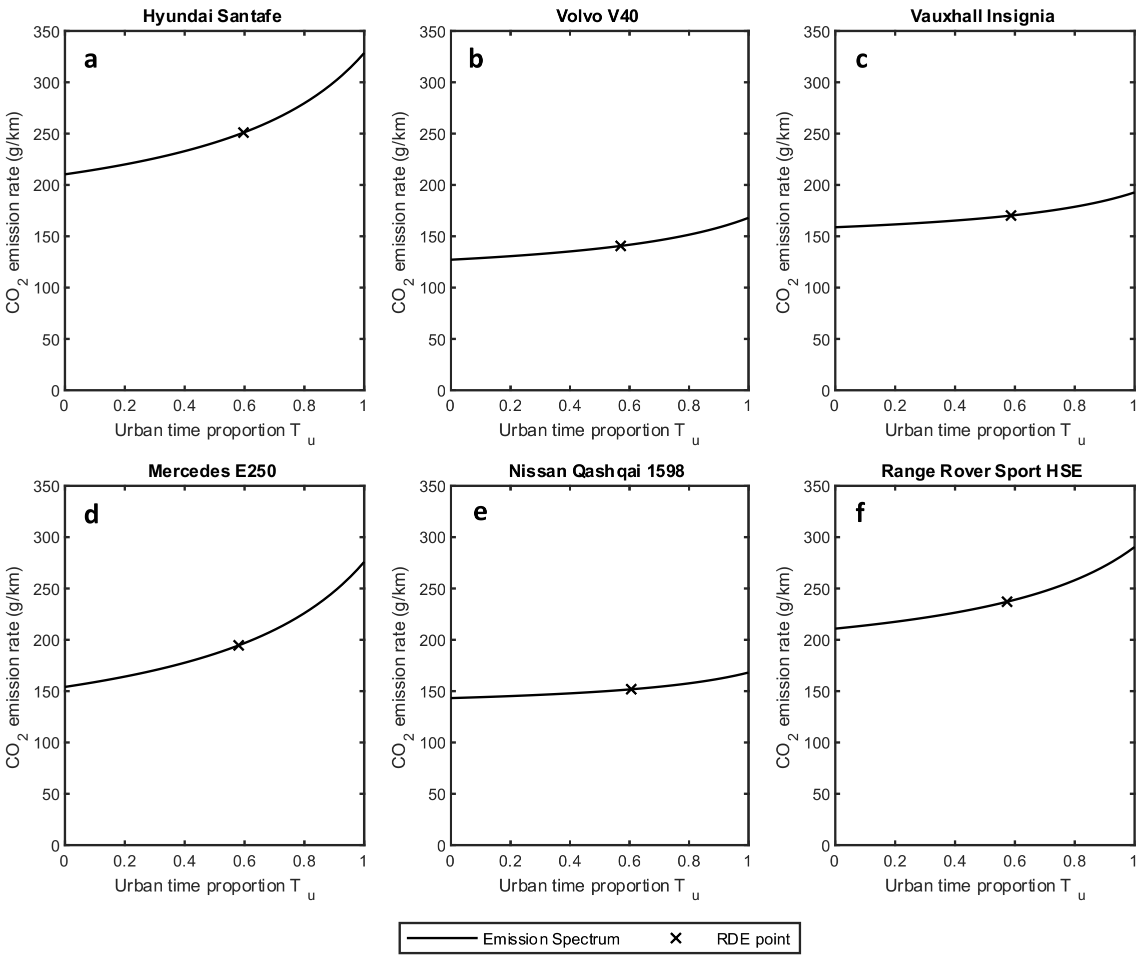

Supplementary Fig. 5 – RLE CO_2 emissions spectrum for Euro 5 Vehicles (Part 2). a-f, Hyundai Santafe (**a**), Volvo V40 (**b**), Vauxhall Insignia (**c**), Mercedes E250 (**d**), Nissan Qashqai 1598 (**e**), Range Rover Sport HSE (**f**). The lines represent variation of the CO_2 emission rate with increasing urban time proportion (for $T_u \in [0,1]$), and the cross-scatter points represent the recorded value at the RDE time proportion, where $T_u \approx 0.6$.

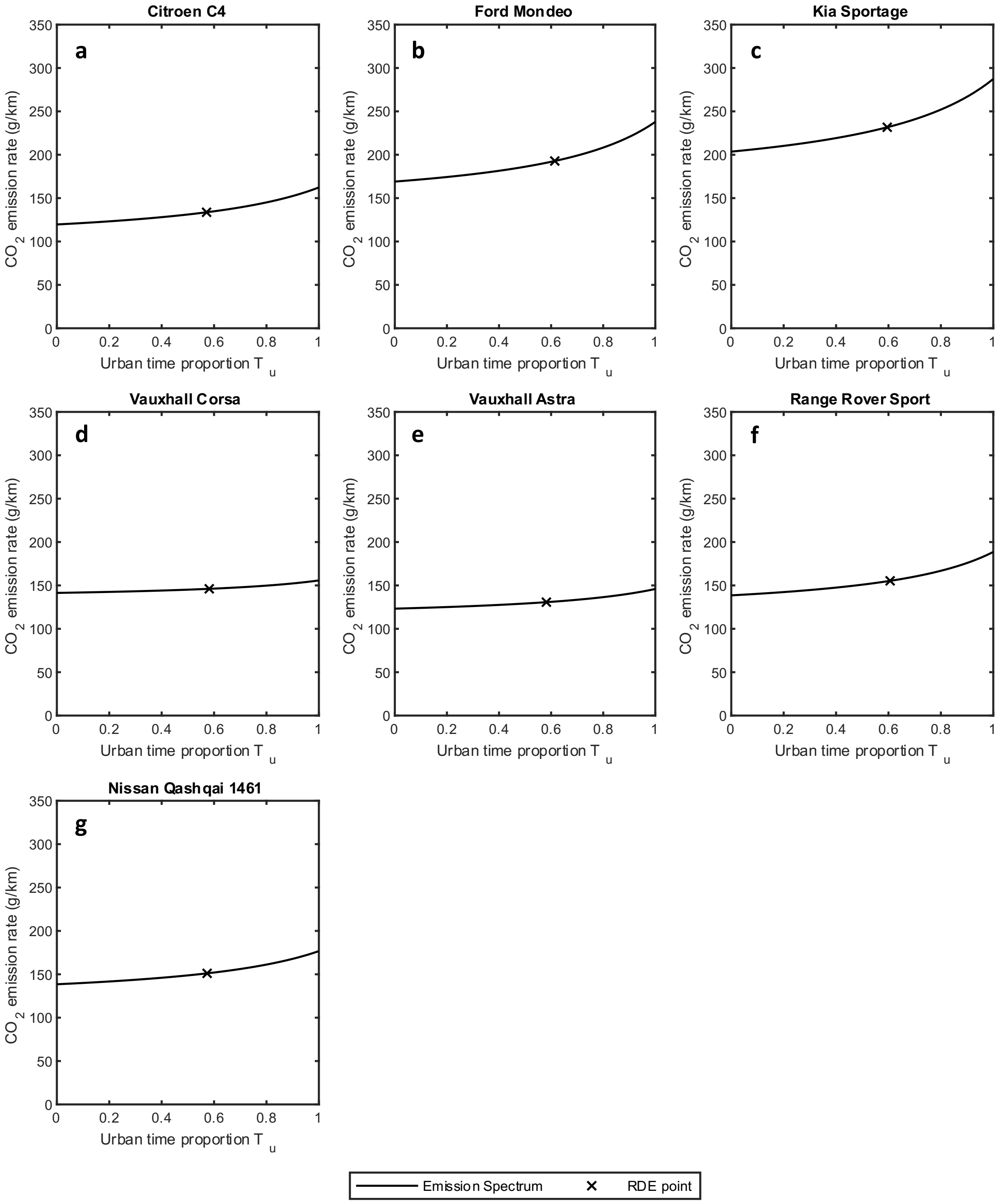

Supplementary Fig. 6 – RLE CO_2 emissions spectrum for Euro 5 Vehicles (Part 3). a-g, Citroen C4 (**a**), Ford Mondeo (**b**), Kia Sportage (**c**), Vauxhall Corsa (**d**), Vauxhall Astra (**e**), Range Rover Sport (**f**), Nissan Qashqai 1461 (**g**). The lines represent variation of the CO_2 emission rate with increasing urban time proportion (for $T_u \in [0,1]$), and the cross-scatter points represent the recorded value at the RDE time proportion, where $T_u \approx 0.6$.

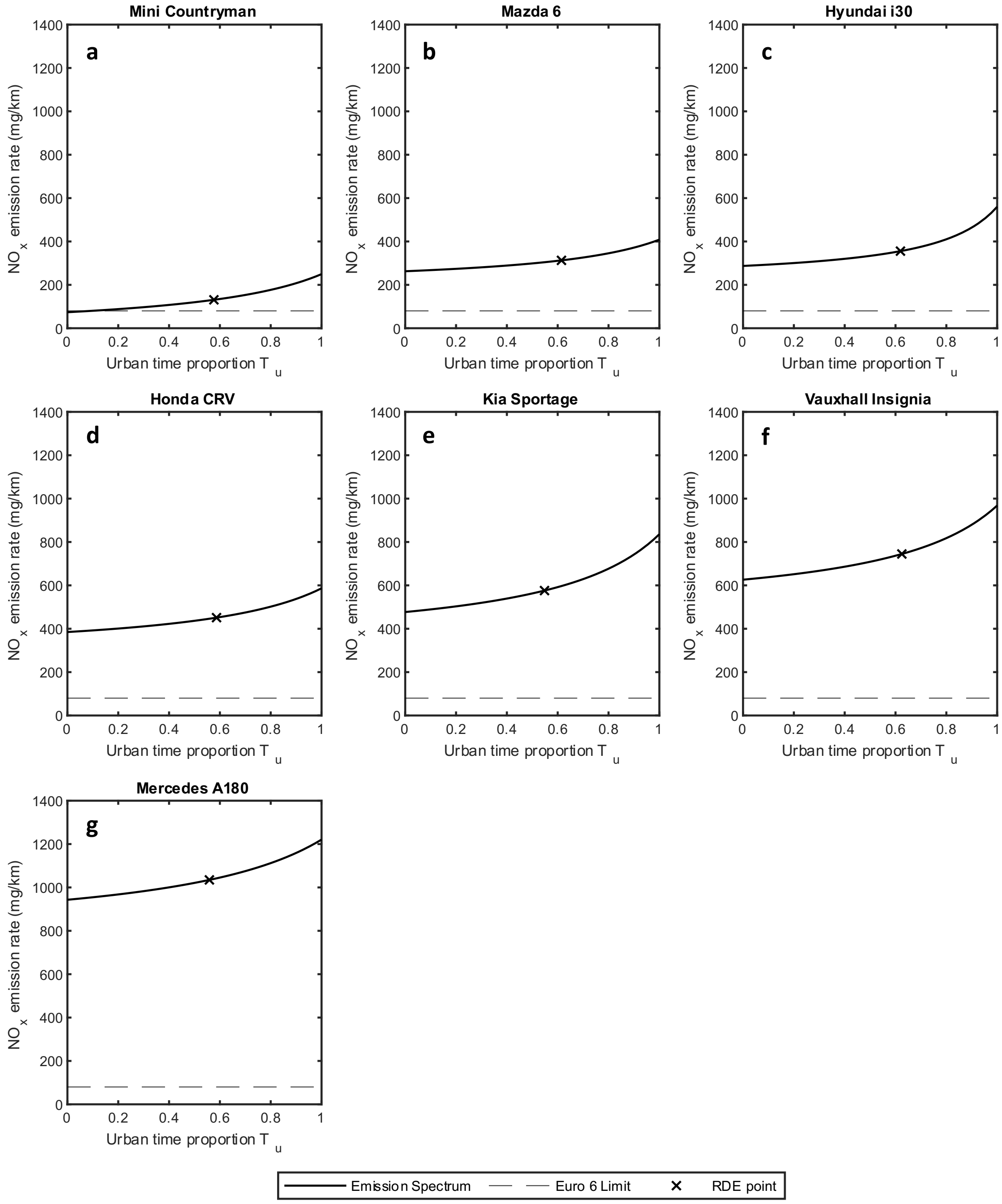

Supplementary Fig. 7 – RLE NO_x emissions spectrum for Euro 6 Vehicles under Group 1: Increasing NO_x emission rate with greater urban time proportion. a-g, Mini Countryman (**a**), Mazda 6 (**b**), Hyundai i30 (**c**), Honda CRV (**d**), Kia Sportage (**e**), Vauxhall Insignia (**f**), Mercedes A180 (**g**). The solid lines represent variation of the NO_x emission rate with increasing urban time proportion (for $T_u \in [0,1]$), the dashed lines represent the Euro 6 NO_x limit at 80 mg/km, and the cross-scatter points represent the recorded value at the RDE time proportion, where $T_u \approx 0.6$.

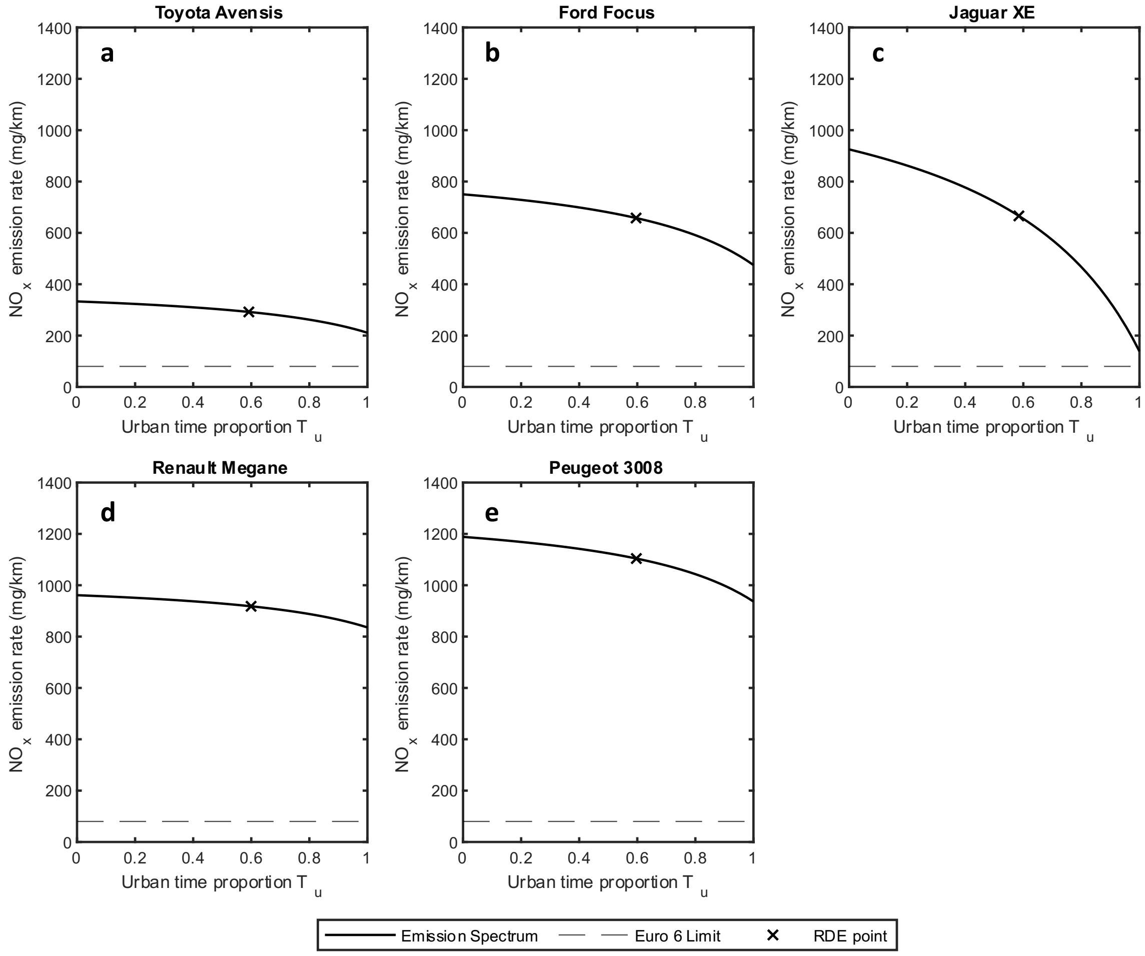

Supplementary Fig. 8 – RLE NO_x emissions spectrum for Euro 6 Vehicles under Group 2: Decreasing NO_x emission rate with greater urban time proportion. a-e, Toyota Avensis (**a**), Ford Focus (**b**), Jaguar XE (**c**), Renault Megane (**d**), Peugeot 3008 (**e**). The solid lines represent variation of the NO_x emission rate with increasing urban time proportion (for $T_u \in [0,1]$), the dashed lines represent the Euro 6 NO_x limit at 80 mg/km, and the cross-scatter points represent the recorded value at the RDE time proportion, where $T_u \approx 0.6$.

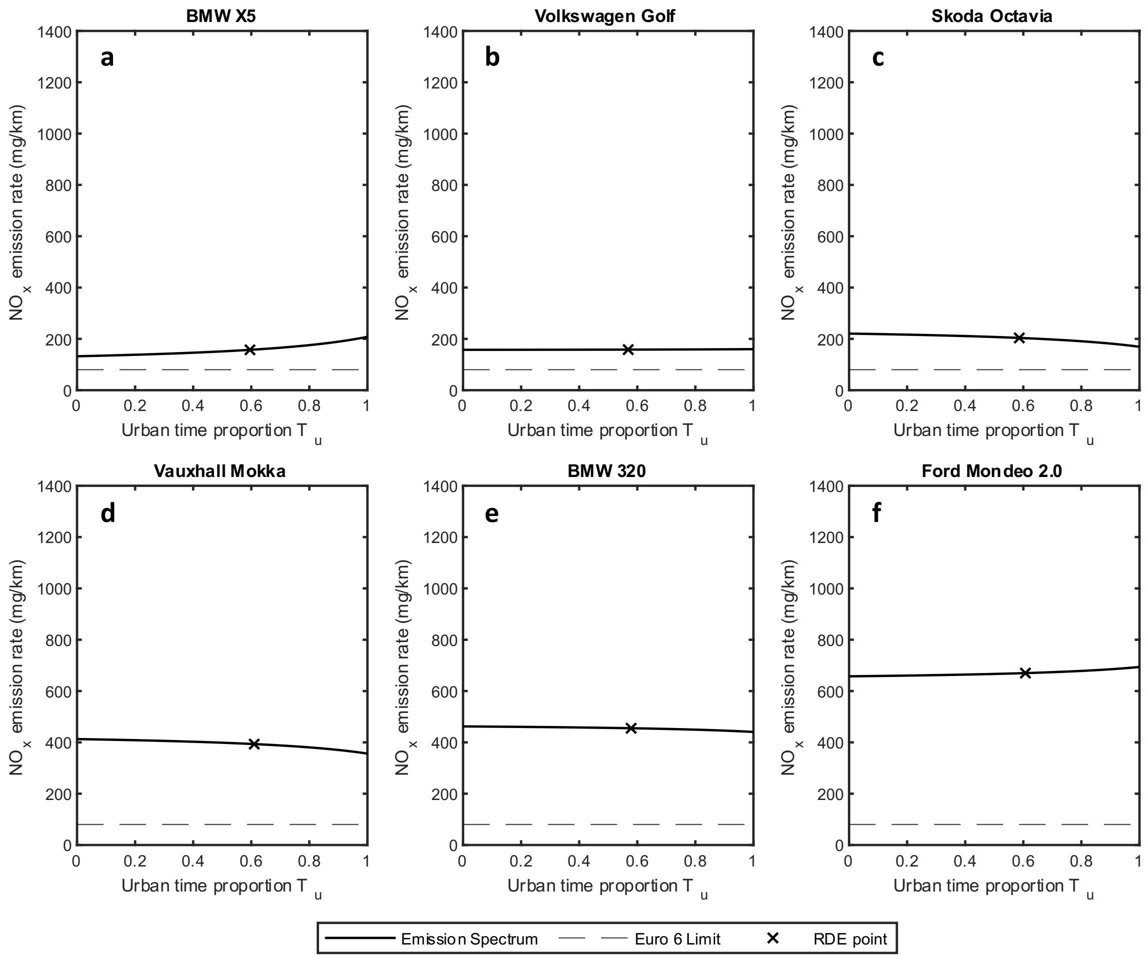

Supplementary Fig. 9 – RLE NO_x emissions spectrum for Euro 6 Vehicles under Group 3: NO_x emission rate independent of urban time proportion (with less than 100 mg/km difference). a-f, BMW X5 (**a**), Volkswagen Golf (**b**), Skoda Octavia (**c**), Vauxhall Mokka (**d**), BMW 320 (**e**), Ford Mondeo 2.0 (**f**). The solid lines represent variation of the NO_x emission rate with increasing urban time proportion (for $T_u \in [0,1]$), the dashed lines represent the Euro 6 NO_x limit at 80 mg/km, and the cross-scatter points represent the recorded value at the RDE time proportion, where $T_u \approx 0.6$.

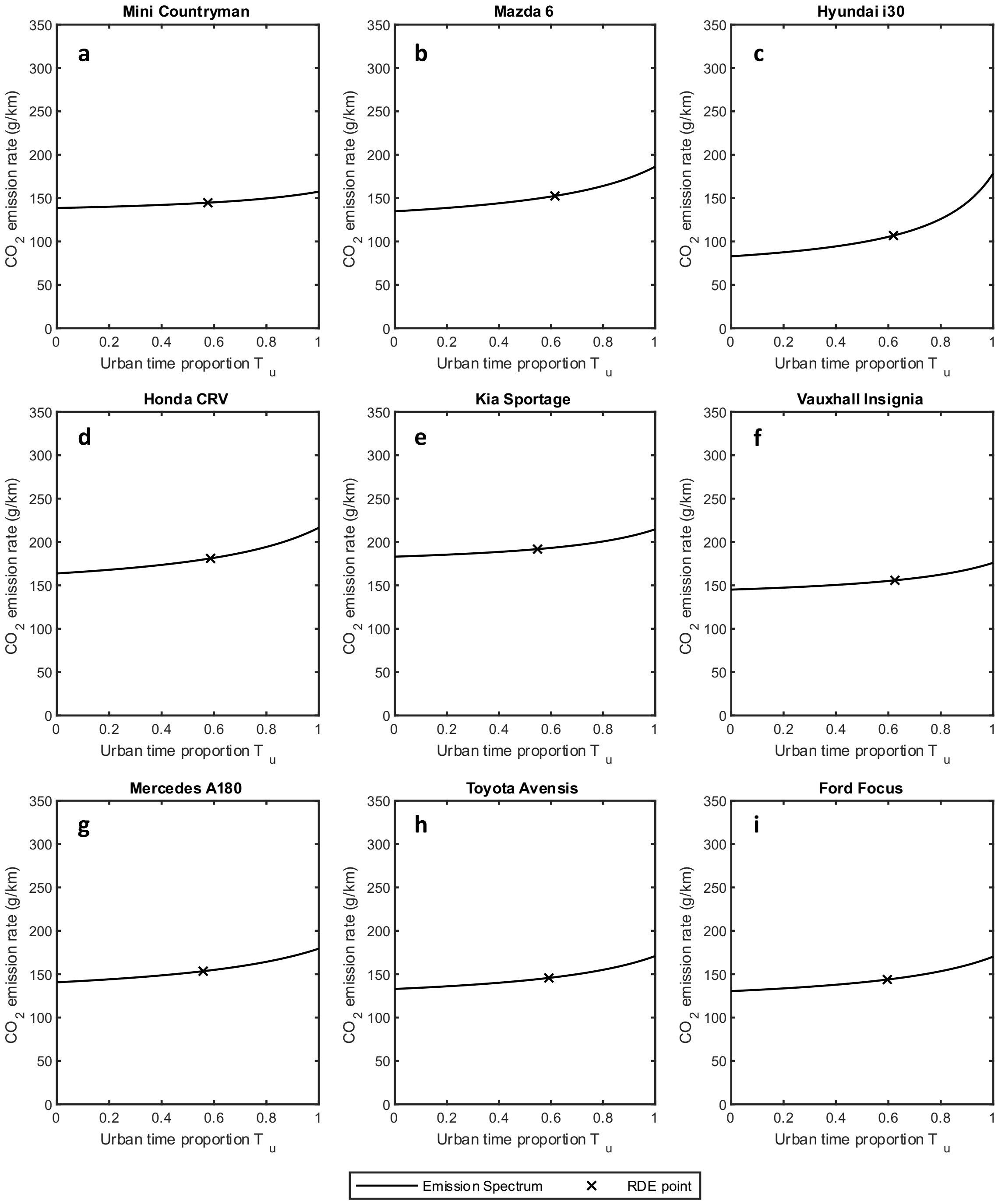

Supplementary Fig. 10 – RLE CO_2 emissions spectrum for Euro 6 Vehicles (Part 1). a-i, Mini Countryman (**a**), Mazda 6 (**b**), Hyundai i30 (**c**), Honda CRV (**d**), Kia Sportage (**e**), Vauxhall Insignia (**f**), Mercedes A180 (**g**), Toyota Avensis (**h**), Ford Focus (**i**). The lines represent variation of the CO_2 emission rate with increasing urban time proportion (for $T_u \in [0,1]$), and the cross-scatter points represent the recorded value at the RDE time proportion, where $T_u \approx 0.6$.

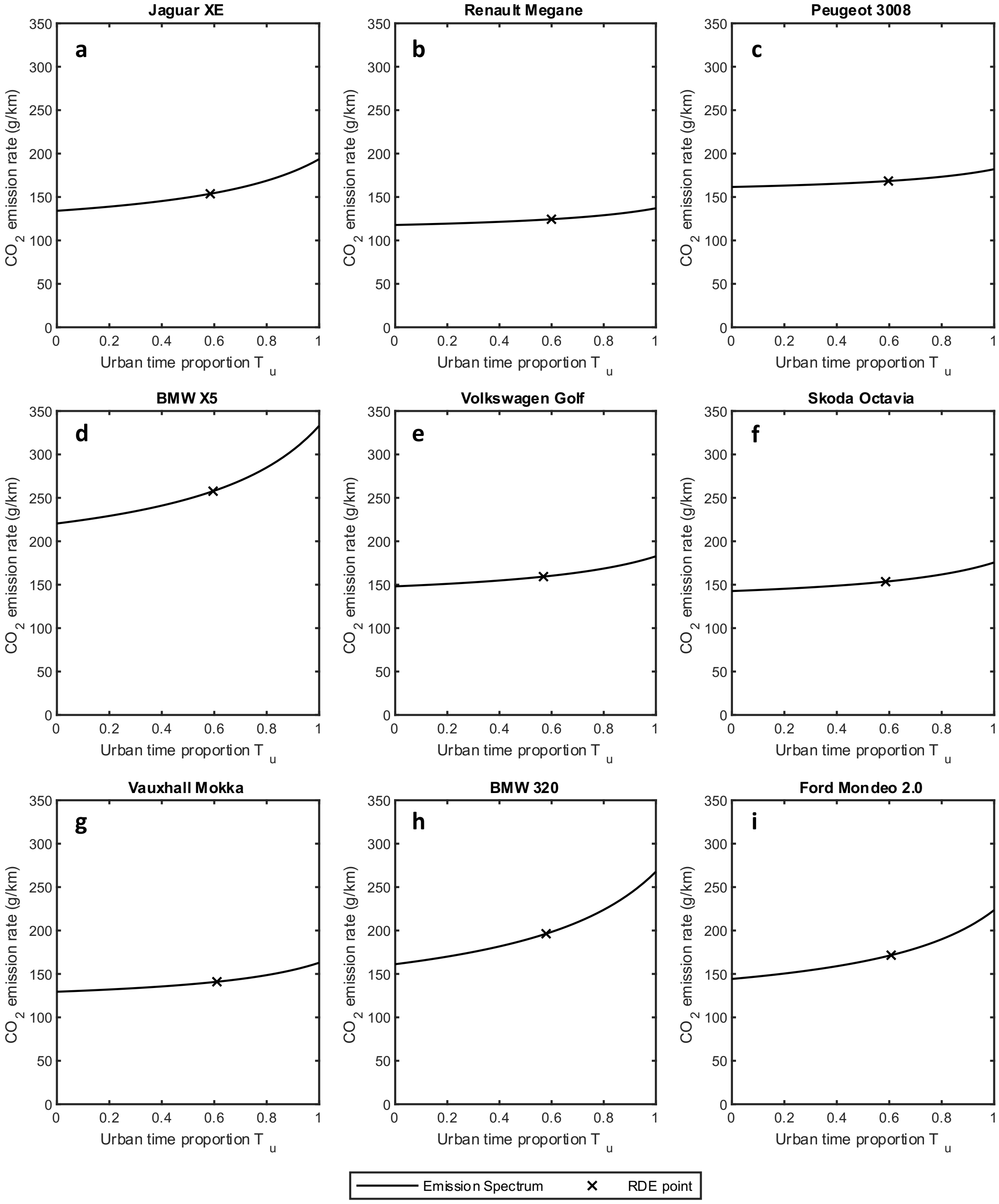

Supplementary Fig. 11 – RLE CO_2 emissions spectrum for Euro 6 Vehicles (Part 2). a-i, Jaguar XE (**a**), Renault Megane (**b**), Peugeot 3008 (**c**), BMW X5 (**d**), Volkswagen Golf (**e**), Skoda Octavia (**f**), Vauxhall Mokka (**g**), BMW 320 (**h**), Ford Mondeo 2.0 (**i**). The lines represent variation of the CO_2 emission rate with increasing urban time proportion (for $T_u \in [0,1]$), and the cross-scatter points represent the recorded value at the RDE time proportion, where $T_u \approx 0.6$.
